# Birth without intervention in women with severe mental illness: cohort study

**DOI:** 10.1192/bjo.2022.24

**Published:** 2022-02-24

**Authors:** Clare Taylor, Robert Stewart, Rod Gibson, Dharmintra Pasupathy, Hitesh Shetty, Louise Howard

**Affiliations:** Department of Psychological Medicine, Institute of Psychiatry, Psychology & Neuroscience, King's College London, UK; Department of Psychological Medicine, Institute of Psychiatry, Psychology & Neuroscience, King's College London, UK; and South London and Maudsley NHS Foundation Trust, London, UK; Rod Gibson Associates Ltd, London, UK; Reproduction and Perinatal Centre, Faculty of Medicine and Health, University of Sydney, Australia; and Department of Women and Children's Health, King's College London, UK; South London and Maudsley NHS Foundation Trust, London, UK; Section of Women's Mental Health, Department of Health Service and Population Research, Institute of Psychiatry, Psychology & Neuroscience, King's College London, UK; and South London and Maudsley NHS Foundation Trust, London, UK

**Keywords:** Schizophrenia, bipolar affective disorders, perinatal psychiatry, birth without intervention, epidemiology

## Abstract

The rate of normal birth outcomes (i.e. full-term births without intervention) for women with severe mental illness (SMI – psychotic and bipolar disorders) is not known. We examined rates of birth without intervention (spontaneous labour onset, spontaneous vaginal delivery without instruments, no episiotomy and no indication of pre- or post-delivery anaesthesia) in women with SMI (584 pregnancies) compared with a control population (70 942 pregnancies). Outcome ratios were calculated standardising for age. Women with SMI were less likely to have a birth without intervention (29.5%) relative to the control population (36.8%) (standardised outcome ratio 0.74, 95% CI 0.63–0.87).

Research shows that most women want to give birth with minimum intervention.^1^ Owing to increasing rates of birth interventions, which confer some risk, there has been a move towards promoting less medicalised birth.^2,3^ Research from Australia has investigated rates and predictors of ‘birth without intervention’ but healthy outcomes have not been investigated in women with psychotic or bipolar disorders (herein termed severe mental illness, SMI).^4,5^ Using linked mental healthcare and hospital admissions data, we investigated rates of having a birth without intervention (also termed ‘normal birth’) in women with SMI. We also stratified by admission to acute psychiatric care, exposure to medication in pregnancy, and affective and non-affective SMI.

## Method

The South London and Maudsley NHS Foundation Trust (SLAM) provides mental healthcare to around 1.2 million residents of four London boroughs, and SLAM's Clinical Record Interactive Search (CRIS) platform provides de-identified copies of electronic records for research use,^6^ linked with Hospital Episode Statistics (HES), which provide national statistical data on all National Health Service (NHS) hospital care in England, including maternity data.^7^ CRIS was approved for research by Oxfordshire Research Ethics Committee C (reference 18/SC/0372).

We used HES data to identify live births and still-births at 24 weeks or over from January 2007 to April 2013 among women receiving care from SLAM at any point from 6 months before to 6 weeks after the delivery who had an SMI diagnosis based on ICD-10 diagnoses F20, F22, F23, F25, F28, F29 (schizophrenia and related disorders, schizoaffective disorders and delusional disorders), F30, F31 (mania and bipolar affective disorders), F32.3, F33.3 (psychotic depression) or F53.1 (puerperal psychosis), excluding SMI diagnosed after the index pregnancy or secondary to an organic disorder.^8^ Pregnancies were dated using an algorithm described previously.^8,9^ We also identified from HES data all live deliveries and still-births at 24 weeks or over from January 2007 to March 2013 from three local providers of obstetric care and used these as a reference population. Commencement of pregnancy and delivery data were ascertained using previously developed algorithms.^9,10^

The outcome, birth without intervention, used a HES-designed composite adapted from the Maternity Care Working Party definition of normal birth, comprising spontaneous labour onset, spontaneous delivery (without instruments), no episiotomy and no mention of either pre- or post-delivery general or regional anaesthesia.^1,10^ This was derived from hospital procedure codes and HES maternity data. Covariates comprised: maternal age at delivery, acute mental healthcare in the 2 years before pregnancy (in-patient and/or home treatment team referral^8,11^), psychotropic medication use in pregnancy (regular antipsychotic, mood stabiliser and/or antidepressant) and affective and non-affective SMI.^8,12^ Non-affective SMI comprised diagnoses of schizophrenia, delusional disorders, acute and transient psychoses, schizoaffective disorders or other non-organic psychoses prior to pregnancy (F20, F22, F23, F25, F28, F29). Affective SMI comprised bipolar affective disorder, psychotic depression and previous postpartum psychosis before the index pregnancy (F30, F31, F32.3, F33.3, F53.1). We also defined preterm birth (<37 weeks from HES-recorded gestational age at birth or ICD-10 codes O601 or O603).

Analyses were carried out using STATA version 13 for Windows. The unit of analysis was birth rather than patient. Standardised outcome ratios (‘birth without intervention’ ratios) were calculated using indirect standardisation against the local population by age strata (<25, 25–29, 30–34, 35+ years) for the whole sample and excluding preterm births. Analyses focused on births at term were then stratified by covariates. This was decided *a priori*, to remove effect modification by preterm births within strata. Birth episodes in the comparison population without HES data on maternal age at delivery were excluded.

## Results

Of 79 621 birth episodes extracted (for 65 330 women), 70 942 had maternal age data and 584 were in women with SMI; mean age at delivery was 31.7 years (s.d. = 6.0 years), 287 (49.1%) had affective diagnoses, 236 (40.4%) had been admitted to acute mental healthcare within the previous 2 years and 438 (75.0%) received psychotropic medication in pregnancy. The SMI cohort had a lower occurrence of normal birth (*n* = 172, 29.5%) than the local population (*n* = 29 093, 36.8%) and higher occurrence of preterm birth (*n* = 76, 13.0% *v*. *n* = 3989, 5.1%). The standardised ‘birth without intervention’ ratio was 0.73 (95% CI 0.62–0.85) for the whole sample; restricted to births at term it was 0.74 (95% CI 0.63–0.87) (Table 1). Stratified analyses did not indicate effect modification by diagnosis, acute mental health admission or medication.

**Table 1:** Birth without intervention in women with severe mental illness (SMI) and control cohorts stratified by age

	Standardised ‘birth without intervention’ ratios, excluding preterm births (95% CI)
Standardised by maternal age	0.74 (0.63–0.87)
Standardised by maternal age and year of delivery	0.74 (0.63–0.87)
Stratified analyses, standardised by maternal age	
Affective SMI (*n* = 71)	0.69 (0.54–0.87)
Non-affective SMI (*n* = 82)	0.79 (0.63–0.98)
Admitted in the 2 years prior to pregnancy (*n* = 68)	0.79 (0.61–1.00)
Not admitted in the 2 years prior to pregnancy (*n* = 85)	0.71 (0.57–0.88)
Psychotropic medication in pregnancy (*n* = 108)	0.72 (0.59–0.67)
No psychotropic medication in pregnancy (*n* = 45)	0.79 (0.58–1.06)

## Discussion

Women with a history of SMI were less likely to have a birth without intervention than a reference population delivering babies in the same hospitals. Rates of birth without intervention in our reference population were lower than a previously reported national average of 40.1% but within the cited between-trust range of 26.0–51.1% (2010–2011)^10^ and similar to national rates from the National Maternity and Perinatal Audit Clinical Report (2016–17) of 36.9%.^4^

Study strengths included use of a standard methodology for processing HES maternity data, including the ‘birth without intervention’ indicator, and a large local reference sample, supporting external validity. Limitations include potential confounding by factors such as ethnicity, smoking, socioeconomic status and trauma, which cannot be accounted for using standardised ratios, in addition to limitations in depth of data on mediating processes, including patient preferences. Although we stratified by psychotropic medication and severity of the psychiatric illness, our study was unlikely to be adequately powered to detect these effects. The standardisation approach did not account for within-individual clustering due to multiple pregnancies, although this would not affect estimates. Also, owing to lower fertility rates in women with SMI, those who become pregnant may be a healthier group, potentially falsely increasing their rates of normal birth relative to the background population.^13^

This is the first report on rates of healthy birth outcomes in women with SMI. In a population-based sample of women in Australia, probability of birth without intervention was reduced in women who were primiparous, had a history of Caesarean section, were older, gave birth to babies at more advanced gestational age and other health conditions in pregnancy, including diabetes and hypertension.^5^ Probability increased if women lived outside major urban areas; modifiable factors identified associated with birth without intervention included having freedom of movement throughout labour and continuity of care in labour and birth. Reasons women with SMI might have lower rates of birth without intervention, include increased social and medical comorbidities compared with the background population, poorer health behaviours (e.g. smoking), exposure to psychotropic medications, and inequalities in perinatal care also require further investigation.^6,8^ Drivers of intervention during birth may be different in the population with and without SMI; for example, rates of anaesthesia desired by the woman and acceptance of acute interventions such as forceps or vacuum might differ and could be disentangled in future research. Women with SMI have high rates of Caesarean section, preterm birth and other adverse neonatal outcomes,^14^ increasing need for acute interventions in labour. However, the effects of stigma or anxiety on rates of interventions are unknown and require further evaluation. Recently, attention has been drawn to the pre-conception period as a window of opportunity to promote healthy perinatal outcomes in women with SMI.^15^ Targets identified for intervention included sleep, social inclusion and pre-conception mental health, as well as diet and physical activity, reducing smoking, alcohol and substance use and addressing safeguarding issues such as domestic abuse. Women with SMI may welcome a paradigm shift in their maternity care by focusing on how to optimise their pregnancy and birth outcomes.

## Data Availability

Data that support the findings of this study are available from the corresponding author on reasonable request.
